# Telitacicept in refractory juvenile generalized myasthenia gravis: a case report and collective analysis

**DOI:** 10.3389/fimmu.2026.1857027

**Published:** 2026-06-08

**Authors:** Yanhong Sun, Guiling Liu, Hongwei Zhang, Kai Ma

**Affiliations:** 1 Neurology Department, Children’s Hospital Affiliated to Shandong University, Jinan, Shandong, China; 2 Neurology Department, Jinan Children’s Hospital, Jinan, Shandong, China

**Keywords:** corticosteroid, generalized myasthenia gravis (gMG), juvenile, myasthenia gravis, telitacicept

## Abstract

**Background:**

Refractory or corticosteroid-dependent juvenile generalized myasthenia gravis (gMG) remains a clinical challenge, given the scarcity of available regimens and prominent corticosteroid-related adverse effects. Telitacicept, a dual inhibitor of B-cell activating factor (BAFF) and a proliferation-inducing ligand (APRIL), has demonstrated efficacy in adult gMG, whereas clinical data in pediatric patients remains limited.

**Objective:**

To evaluate the clinical response, tolerability, and potential corticosteroid-sparing benefit of telitacicept in juvenile refractory gMG.

**Methods:**

We report one new case of juvenile refractory gMG treated with telitacicept and review three previously reported pediatric gMG cases. Collective analysis was performed to evaluate key outcomes, including changes in Myasthenia Gravis Activities of Daily Living (MG-ADL) score, Quantitative Myasthenia Gravis (QMG) score, corticosteroid dose reduction, and adverse events.

**Results:**

In the index case, telitacicept reduced the MG-ADL score from 3 to 0 and the QMG score from 10 to 3 by week 12. By week 32, the MG-ADL score remained at 0, while the QMG score further decreased to 2, accompanied by a 41.6% reduction in corticosteroid dose. The patient developed mild injection-site erythema and pruritus on only one occasion, which resolved spontaneously. She also had a mild upper respiratory tract infection that resolved within one week following symptomatic treatment. Collective analysis of the four included patients (age range: 7–17 years) showed that three cases (Cases 1, 2, and 4) achieved marked improvement (≥3-point reduction in the MG-ADL score) at 3 months. By 5–6 months, all patients presented sustained clinical improvement with a ≥2-point reduction in the MG-ADL score. No severe adverse events were reported.

**Conclusion:**

Telitacicept may be of clinical value and well-tolerated, and may exert a corticosteroid-sparing benefit in juvenile refractory gMG, providing preliminary real−world clinical observations for this understudied population. Further large-scale prospective studies are warranted to confirm these findings.

## 1 Introduction

Myasthenia gravis (MG), an autoimmune disorder of the neuromuscular junction (NMJ), is characterized by autoantibodies and presents clinically with fluctuating skeletal muscle weakness and pathological fatigability (1). The most common antibodies (Abs) associated with MG are those directed against the acetylcholine receptor (AChR). In the pediatric population, ocular MG represents the most common clinical phenotype (2). However, in a subset of patients, disease progression occurs with the extension of muscle weakness beyond the extraocular muscles, leading to generalized MG (gMG) (3). First-line therapy mainly comprises acetylcholinesterase inhibitors and corticosteroids (1). Second-line nonsteroidal immunosuppressive agents, such as mycophenolate mofetil (MMF) and tacrolimus (TAC), are commonly used to reduce corticosteroid exposure and prevent relapse (1). Plasma exchange (PLEX) and intravenous immunoglobulin (IVIg) are usually reserved for patients with acute exacerbations (1). However, 10%–20% of patients exhibit an inadequate response or intolerance to these conventional agents, leading to refractory MG (4, 5). Conventional immunotherapy, particularly corticosteroids, often causes systemic adverse effects that may affect the pediatric growth and development, which is a serious concern. Telitacicept, a novel recombinant fusion protein, consists of the transmembrane activator and calcium modulator and cyclophilin ligand interactor (TACI) fused to the Fc portion of human immunoglobulin G (IgG) (6, 7). It simultaneously inhibits B lymphocyte stimulator (BLyS, also known as BAFF) and a proliferation-inducing ligand (APRIL), thereby suppressing B-cell proliferation, activation, and differentiation, and lowering pathogenic autoantibody levels, which in turn alleviates disease severity (6, 7). Furthermore, its phase III clinical trial in MG has shown promising results, with real-world studies supporting its efficacy and safety (8–14). In May 2025, telitacicept was approved by the National Medical Products Administration (NMPA) in China as an add-on therapy for adult patients with AChR-Ab-positive gMG. However, clinical data on telitacicept in juvenile gMG remain very limited, with only three cases reported in the literature to date (see 3.2 Collective Analysis of Four Case) (9, 13). We report a case of juvenile gMG treated with telitacicept and review previously published pediatric cases. This article summarizes the clinical response and tolerability profile of telitacicept in juvenile gMG, contributing preliminary clinical observations in this understudied population.

## 2 Methods

### 2.1 Data collection and clinical assessment

Clinical data including demographics, medical history, treatment regimens, laboratory values, and electrophysiological studies were collected retrospectively. Clinical status was assessed at baseline and during follow-up using the MG-associated Activities of Daily Living (MG-ADL), Quantitative Myasthenia Gravis (QMG), and Myasthenia Gravis Quality of Life 15-Item Scale (MG-QoL15).MG-QoL15 assessments at an earlier stage were not completed due to objective reasons. The patient was followed regularly with clinical and laboratory monitoring until the most recent visit.

### 2.2 Literature search and data extraction

We systematically searched PubMed, Embase, Web of Science, Wanfang Data Knowledge Service Platform, China National Knowledge Infrastructure (CNKI), and Chinese Medical Journal Full-Text Database through February 2026 using the keywords “telitacicept”, “myasthenia gravis”, “generalized myasthenia gravis”, and “juvenile generalized myasthenia gravis”. Inclusion criteria were age ≤18 years, confirmed gMG, telitacicept treatment, and available clinical outcomes. Data on baseline characteristics, treatment, and outcomes were extracted independently by two reviewers.

## 3 Case presentation

### 3.1 Clinical features and treatment outcomes of the index case

**Figure 1:**
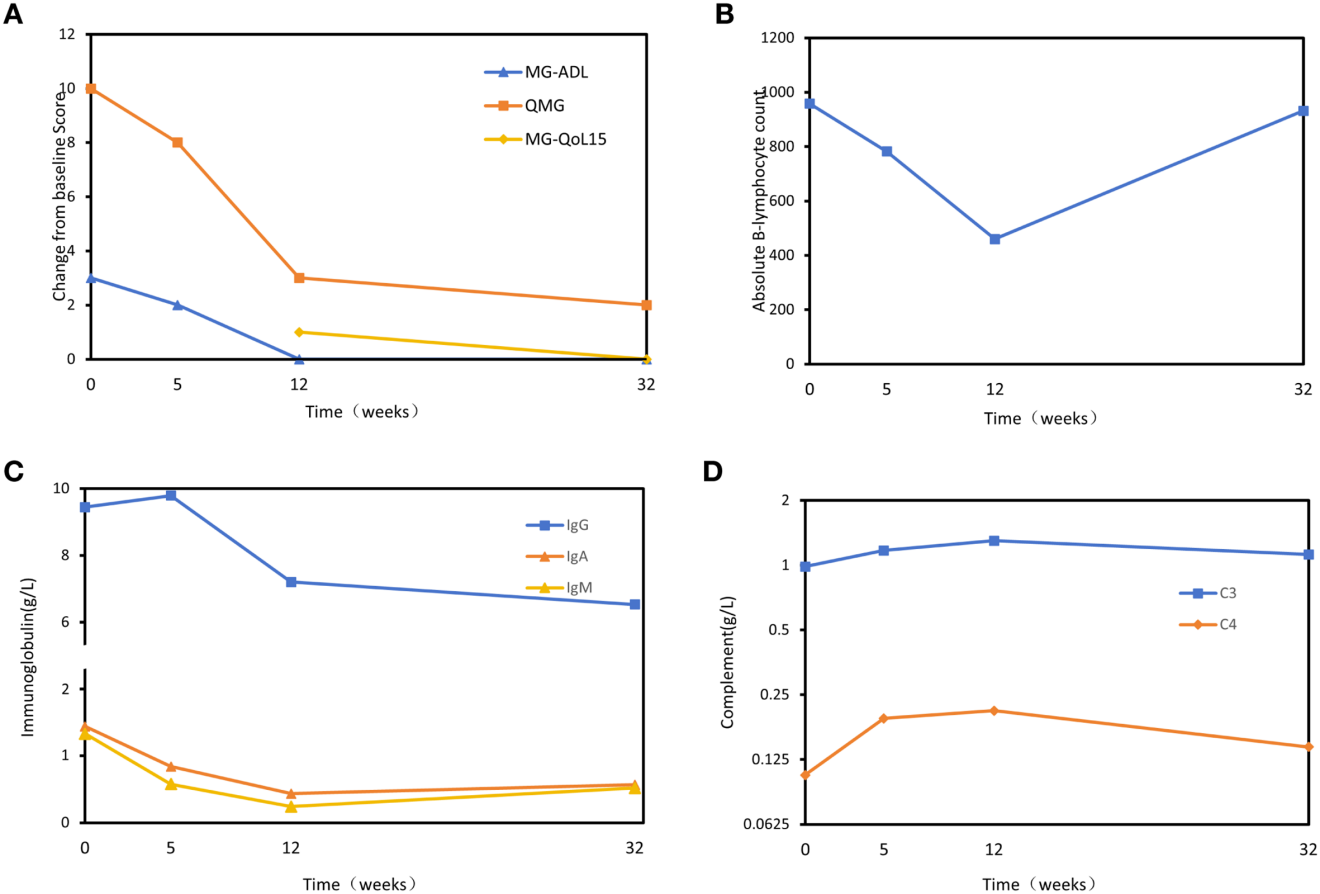
Clinical and immunological changes during follow-up from baseline to week 32. **(A)** Changes from baseline to week 32 in MG-ADL, QMG, and MG-QoL15 scores. **(B)** Changes from baseline to week 32 in absolute B-lymphocyte count. **(C)** Changes from baseline to week 32 in IgG, IgA, and IgM. **(D)** Changes from baseline to week 32 in serum complement C3 and C4. MG-ADL, myasthenia gravis-specific activities of daily living scale; QMG, quantitative myasthenia gravis score; MG-QoL15, myasthenia gravis quality of life 15-item scale; IgA, immunoglobulin A; IgG, immunoglobulin G; IgM, immunoglobulin (M) Laboratory Reference Range for Indicators: absolute B-lymphocyte count: 247–578 cells/μL; IgG:7.0–15.5 g/L; IgM:0.29–0.41 g/L; IgA: 0.58–2.91 g/L; C3:0.83–1.61 g/L; C4: 0.13–0.41 g/L.

An 11-year-old female presented to our hospital in June 2024. She had a 4–5-year history of ptosis prior to presentation. Two months before admission, she experienced clinical worsening with progressive, fluctuating dysphagia, hoarseness, and limb weakness. Diagnostic evaluation showed positive results on serological testing for AChR-Ab (1.44 nmol/L, tested by ELISA, reference value ≤ 0.45 nmol/L), prostigmin test, and the 3 Hz repetitive nerve stimulation (RNS) test. Chest computed tomography (CT) showed no thymic abnormalities. She was therefore diagnosed with AChR-Ab-positive gMG, with MG-ADL score of 11, corresponding to Myasthenia Gravis Foundation of America (MGFA) Class IIIb. This also represented her maximum disease severity across the entire disease course. She was started on prednisone at a dose of 30 mg once daily, alongside pyridostigmine at a dose of 30 mg three times daily; she also received a single IVIg infusion at a dose of 2 g/kg. In July 2024, the patient’s dysphagia, hoarseness, and limb weakness improved, and her MG-ADL score decreased by 6 points. As ptosis persisted, pyridostigmine was increased to 60 mg three times daily. Although initial symptoms improved, persistent ptosis and generalized weakness persisted over the subsequent 12 months, and attempts at corticosteroid tapering were unsuccessful. She also developed prominent Cushingoid features, including moon face and hirsutism. Because of an unsatisfactory clinical response, the patient was readmitted to our hospital in July 2025. At that time, her QMG score was 10 and MG-ADL score was 3, consistent with MGFA Class IIb, representing the baseline MGFA classification prior to telitacicept initiation. Her MG-ADL score of 3 was mainly attributed to the item related to persistent ptosis. The patient was classified as corticosteroid-dependent, refractory juvenile gMG due to insufficient symptom control and corticosteroid-related adverse effects despite ≥12 months of standard therapy. The patient and her guardian declined conventional nonsteroidal oral immunosuppressants because of safety concerns. After excluding contraindications and obtaining written informed consent from her guardian, telitacicept was administered subcutaneously at a dose of 160 mg once weekly for 12 weeks, with the dosing interval subsequently extended based on clinical response. Concurrently, prednisone was switched to an equipotent dose of oral methylprednisolone to minimize adverse effects. Telitacicept yielded a favorable clinical response. Clinical improvement was evident as early as 3 weeks after treatment initiation, especially in ptosis and limb muscle strength. At week 5, the MG-ADL score was 2 and the QMG score was 8. By week 12, the QMG score had decreased by 7 points, with an MG-ADL score of 0 and an MG-QoL15 score of 1. By week 32, both MG-ADL and MG-QoL15 scores remained at 0, while the QMG score was 2; the corticosteroid dose was reduced by 41.6%, and Cushingoid features improved accordingly. **Figure 1A** demonstrates the changes in MG-ADL, QMG, and MG-QoL15 scores. Absolute B−lymphocyte count, IgG, IgA, IgM, and complement C3/C4 were monitored serially during telitacicept therapy, as depicted in **Figures 1B–D**. Absolute B-lymphocyte counts initially decreased but remained above the lower limit of normal throughout follow-up. Serum IgG, IgM, and IgA levels declined, with nadir values below the normal range. C3 and C4 levels were transiently elevated at baseline but remained within normal limits overall. The patient developed transient mild injection-site erythema and pruritus on only one occasion (resolved spontaneously within 2–3 days) and a mild upper respiratory tract infection that resolved within 1 week following symptomatic treatment with paracetamol, ibuprofen, and cephalosporin antibiotics. These events were self-limiting and did not require telitacicept discontinuation. Notably, exacerbation of extraocular muscle weakness occurred at week 13 of treatment. After prompt infection management and adjustment of the corticosteroid tapering schedule, her symptoms were effectively controlled within 1 week and remained stable thereafter. This transient exacerbation was deemed unrelated to telitacicept and attributed to concurrent infection and overly rapid corticosteroid tapering. No other adverse events were observed. The patient remains asymptomatic and continues maintenance therapy with regular follow-up.

### 3.2 Collective analysis of four cases

After screening, two previously published studies (encompassing 3 juvenile gMG patients) met all inclusion criteria (9, 13). Combined with our index case, the baseline characteristics of these four patients are summarized in **Table 1**, and telitacicept-related clinical outcomes are depicted in **Table 2**. All four patients were female, with a mean age at onset of 13.5 ± 4.4 years (range, 7–17 years), a mean age at treatment initiation of 15.0 ± 2.8 years (range, 11–17 years), and a median disease duration of 16.5 months (IQR, 4.0–37.0). According to MGFA classification, three patients (75.0%) were class IIa and one (25.0%) class IIb. Myasthenic crisis history was not reported in one patient, while the other three had no history of crisis. Anti-AChR-Abs were positive in three patients (75.0%) and negative in one (25.0%), with no thymoma identified in any case. Comorbidities (thyroid disease and diabetes mellitus, two patients each) were present in three of the four patients (75.0%). At baseline, three patients (75.0%) were on oral prednisone (≥ 30 mg/day).Case 1 (our index case) had long-term corticosteroid therapy (> 1 year) with inadequate symptom control and severe corticosteroid-related adverse effects. Her parents declined concomitant nonsteroidal immunosuppressants. Cases 2 and 3 had refractory disease (inadequate respond to ≥ 2 prior immunosuppressive therapies, drug intolerance due to severe adverse effects, frequent relapses during corticosteroid tapering, or dependence on IVIg or plasmapheresis [PE]). The guardians of Case 4 declined corticosteroids and nonsteroidal immunosuppressants due to concerns about potential adverse effects. Overall, all four patients represented a difficult-to-treat population with suboptimal responses, intolerance, or refusal to conventional immunosuppressive therapies.All received telitacicept as add-on therapy: Case 1 (160 mg weekly for the initial 12 weeks, then gradual interval extension), Case 4 (160 mg weekly for the initial 4 weeks, then gradual extension), and Cases 2–3 (160 mg every 2 weeks without adjustment). At 3 months, three patients (Cases 1–2, 4) achieved marked improvement (≥ 3-point reduction in MG-ADL score). By 5–6 months, all patients demonstrated clinical improvement (≥ 2-point reduction in MG-ADL score); Case 3 showed the mildest response (2-point MG-ADL reduction and 16.7% reduction in corticosteroid dose). No serious adverse events were reported in any patient during treatment.

**Table 1:** Baseline characteristics of four juvenile generalized myasthenia gravis patients treated with telitacicept.

Case no.	Authors and Reference	Sex	Age at onset (Y)	Disease duration (M)	MGFA class	Myasthenia crisis	Antibody subtypes	Thymoma	Comorbidities
1	Present case	F	7	48	IIb	No	AChR-Ab	No	Thyroid disease
2	Lin J et al. (9)	F	15	7	IIa	No	AChR-Ab	No	DM, thyroid disease
3	Lin J et al. (9)	F	15	26	IIa	No	AChR-Ab	No	DM
4	Fang Z et al. (13)	F	17	1	IIa	NR	Negative	No	None

AChR-Ab, anti-acetylcholine receptor antibody; DM, diabetes mellitus; F, female; M, months; Y, years; MGFA, Myasthenia Gravis Foundation of America; NR, not reported.

**Table 2:** Treatment and clinical outcomes of individual patients.

Case no.	Pre-telitacicept Immunotherapies	Baseline Prednisone Dosage (mg/d)	Baseline MG-ADL	Baseline QMG	Month 3 Prednisone (mg/d)	Month 3 MG-ADL	Month 3 QMG	Month 6–7 Prednisone (mg/d)	Month 6–7 MG-ADL	Month 6–7 QMG
1	Prednisone + IVIg	30	3	10	20^*^	0	3	17.5^*†^	0^†^	2^†^
2	Prednisone + MMF	0	4	10	0	1	8	0	0	6
3	FK506 + Prednisone + MMF + IVIg + PE	30	3	10	30	2	8	25	1	8
4	Prednisone	30	3	NR	NR	0	NR	NR	NR	NR

*Prednisone dose was converted from an equipotent dose of methylprednisolone. †Data at month 7; data at month 6 for cases 2 and 3.

MG-ADL, Myasthenia Gravis Activities of Daily Living scale; QMG, Quantitative Myasthenia Gravis scale; IVIg, intravenous immunoglobulin; MMF, mycophenolate mofetil; PE, plasmapheresis; FK506, tacrolimus; NR, not reported.

## 4 Discussion

A proportion of pediatric patients with gMG exhibit suboptimal responses to conventional immunosuppressive regimens or are unable to tolerate them, consistent with observations in this study. There is an urgent need for novel therapeutic alternatives for this vulnerable population. Telitacicept, a dual BAFF/APRIL inhibitor, has demonstrated favorable efficacy and safety in adults with gMG (8, 15), which has been further supported by phase III and real-world studies (8–12, 14). However, high-quality clinical evidence in juvenile gMG remains scarce. Herein, we report a detailed pediatric case and review three previously published cases, to evaluate the clinical response and tolerability of telitacicept in juvenile gMG, particularly in refractory or corticosteroid-dependent patients. Our case showed that add-on telitacicept was associated with noticeable clinical improvement in an 11-year-old female with AChR-Ab-positive, corticosteroid-refractory gMG (MGFA Class IIb). By week 12, her MG-ADL score had decreased by 3 points to 0, and the QMG score had decreased by 7 points to 3. At week 32, MG-ADL remained at 0, the QMG score was 2, and corticosteroids were successfully tapered by 41.6%, with no severe adverse events. The observed corticosteroid-sparing effect and tolerable safety profile were generally in line with previous literature (13), which may imply a potential role of telitacicept in clinical practice. Collective analysis of the four cases further suggested that three of the four patients achieved apparent improvement at 3 months, and all patients tended to show a clinical response by 5 to 6 months of treatment, with an acceptable overall safety profile. Mild-to-moderate reductions in absolute B-lymphocyte counts and IgG/IgM/IgA levels were observed, consistent with telitacicept’s dual BAFF/APRIL blockade mechanism and previous phase III and real-world findings (8, 11). The absolute B-lymphocyte counts remained within normal limits, and no severe hypogammaglobulinemia or opportunistic infections occurred, suggesting that these changes represent predictable pharmacodynamic effects rather than major safety concerns. A transient exacerbation of extraocular muscle weakness occurred at week 13, most likely attributable to concomitant infection and overly rapid corticosteroid tapering rather than telitacicept. This highlights the importance of individualized corticosteroid tapering and prompt infection management. Notably, clinical efficacy was also observed in the seronegative patient, suggesting telitacicept may act through non-antibody-mediated pathways. Although non-response to telitacicept has been reported in MG (9), the underlying mechanisms remain poorly understood. Recent evidence suggests that neutrophils drive MG exacerbation via BAFF secretion (16), suggesting that patients with normal or low neutrophil counts may have a poorer response to telitacicept — a hypothesis that warrants further validation. Another B-cell–directed biologic, rituximab (RTX), is commonly employed in the management of juvenile refractory MG (17, 18). However, RTX induces profound and persistent B-cell depletion, which may increase the risk of infections and hypogammaglobulinemia in pediatric patients. In contrast, telitacicept might serve as a potential alternative to RTX in pediatric patients and appear to be better tolerated by the developing immune system. Our case provides 32 weeks of follow-up data suggesting sustained remission and successful corticosteroid tapering, which may partially fill the gap in long-term data for juvenile gMG. Collective analysis of four cases further suggests the consistent clinical improvement and tolerability profile of telitacicept in juvenile gMG. Telitacicept may be of potential value for corticosteroid-dependent or refractory pediatric patients, as it could facilitate corticosteroid tapering and thereby help reduce corticosteroid-related adverse effects. This case series has several limitations. The small sample (only four patients) and lack of a control group limit the robustness of the conclusions. Follow-up duration was relatively short (up to 32 weeks), limiting assessment of long-term outcomes. Some incomplete baseline and follow-up data may somewhat affect the reliability of our findings. Baseline clinical heterogeneity may introduce confounding factors, and enrollment of only female patients limits generalizability to male patients. Furthermore, the optimal dose and dosing interval of telitacicept in pediatric patients remain to be determined. Larger, long-term prospective studies are therefore warranted to confirm the clinical response, tolerability, and optimal dosing regimen of telitacicept in juvenile gMG.

## 5 Conclusion

Telitacicept appears to yield a favorable clinical response and tolerability in juvenile gMG, with potential corticosteroid-sparing benefit, especially in refractory or corticosteroid-dependent patients. Individualized treatment and close clinical monitoring are essential for optimal outcomes. Larger prospective studies with long-term follow-up are warranted to further clarify its clinical response, tolerability, and optimal dosing in pediatric patients.

## Data Availability

The original contributions presented in the study are included in the article/supplementary material. Further inquiries can be directed to the corresponding author.
